# Usefulness of criteria for intraoperative Management of Postoperative Nausea and Vomiting

**DOI:** 10.1186/s40780-022-00242-1

**Published:** 2022-04-04

**Authors:** Satoshi Nagase, Masaharu Imaura, Mizuki Nishimura, Kohei Takeda, Mari Takahashi, Hideki Taniguchi, Tomoyuki Sato, Hiroshi Kanno

**Affiliations:** 1 Department of Pharmacy, Saiseikai Yokohamashi Tobu Hospital, 3-6-1, Shimosueyoshi, Tsurumi-ku, Yokohama-shi, Kanagawa, 230-8765 Japan; 2 Patient Support Center, Saiseikai Yokohamashi Tobu Hospital, 3-6-1, Shimosueyoshi, Tsurumi-ku, Yokohama-shi, Kanagawa, 230-8765 Japan; 3 Department of Anesthesiology, Saiseikai Yokohamashi Tobu Hospital, 3-6-1, Shimosueyoshi, Tsurumi-ku, Yokohama-shi, Kanagawa, 230-8765 Japan

**Keywords:** Apfel simplified score, Postoperative nausea and vomiting, Pharmacist, Anesthesiologist, Criteria, Management

## Abstract

**Background:**

Postoperative nausea and vomiting (PONV) delays postoperative recovery, prolongs hospital stays, and hinders patients’ return to society, thus making it a major cause of increased healthcare costs. It is also the most troubling postoperative complication in female patients undergoing surgery. However, in Japan, guidelines for the management of PONV have not been established, and the management protocol for PONV is left to each institution and anesthesiologist. Therefore, we developed criteria for intraoperative management of PONV.

**Methods:**

In female surgical patients, the usefulness of the criteria was evaluated by comparing the implementation rate of intraoperative management and PONV incidence before and after the establishment of the criteria. An Apfel simplified score (Apfel score) ≥2 was set as an indication for intraoperative management of PONV.

**Results:**

The implementation rate of intraoperative management increased from 91.2 to 96.0% after the introduction of the criteria. In patients with an Apfel score of 2, the intraoperative management implementation rate significantly increased from 81.1 to 94.7% (*p* = 0.016), while PONV incidence significantly decreased from 44.6 to 34.1% after the introduction of the criteria (*p* = 0.040).

**Conclusions:**

The criteria for intraoperative management of PONV increased the implementation rate of intraoperative management and decreased PONV incidence, indicating the usefulness of the criteria.

## Background

Postoperative nausea and vomiting (PONV) occurs in approximately 30% of all surgical patients [1, 2], delays postoperative recovery, prolongs hospital stay, and hinders patients’ return to society, thus making it a major factor that increases healthcare costs [3]. Moreover, it is strongly associated with decreased patient satisfaction with surgery and anesthesia [4]. In female surgical patients, PONV is more uncomfortable than postoperative pain and is considered the most troubling postoperative complication [5]. Thus, PONV management has been strongly recommended in some countries to promote postoperative recovery [1, 6, 7]. This includes avoidance of PONV-inducing factors, such as postoperative opioid analgesics, volatile anesthetics, and inappropriate rehydration, as well as the necessity of using antiemetic agents based on PONV development risk [1].

In Japan, the incidence of PONV is approximately 40% [8], which is higher than the previously mentioned 30% observed in all patients; therefore, it cannot be concluded that PONV management has been sufficiently implemented in the country. A probable reason for this is the lack of established guidelines for PONV management in Japan; the management protocol for PONV is left to each institution and anesthesiologist. Furthermore, as with other factors in Japan, there are fewer approved drugs for PONV than in other countries, resulting in a limited choice.

Therefore, the pharmacists and anesthesiologists at our institution, Saiseikai Yokohamashi Tobu Hospital, collaborated to develop the criteria for intraoperative management of PONV, with the aim of standardizing PONV management and reducing its incidence. In this study, the implementation rate of intraoperative management, as well as PONV incidence, was investigated to evaluate the usefulness of the criteria for intraoperative management.

## Methods

### Activities of pharmacists in perioperative management of PONV

**Fig. 1:**
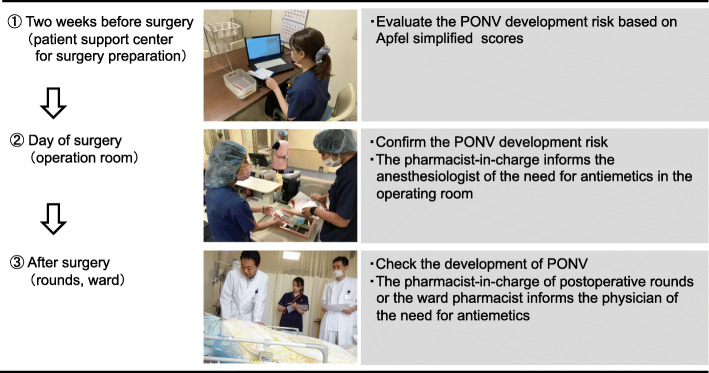
Activities of a pharmacist who engages in PONV management in our institution. PONV: postoperative nausea and vomiting

Figure 1 shows the activities of pharmacists in the perioperative management of PONV in our hospital. A pharmacist evaluated the patients’ PONV development risk based on an Apfel simplified score (Apfel score) [9, 10] upon their visit to the patient support center for surgery preparation 2 weeks before surgery. On the day of surgery, the pharmacist-in-charge in the operating room confirmed the risk of PONV development and proposed the administration of antiemetics to the anesthesiologist based on the criteria for intraoperative management. After surgery, pharmacists who participated in postoperative rounds or resident pharmacists in the ward checked the development of PONV and proposed the administration of antiemetics to physicians in cases where PONV was observed.

### Criteria for intraoperative management of PONV

Pharmacists and anesthesiologists in our institution developed criteria for intraoperative management with the aim of standardizing PONV management and reducing its incidence. An Apfel score of more than 3 was set to be the criteria for intraoperative management of PONV for men, while more than 2 for women, and the antiemetics to be used are dexamethasone, droperidol, or metoclopramide. The pharmacist in charge of the operating room proposed that the anesthesiologist use antiemetics in patients who met the intraoperative criteria. However, the choice of drugs, dosage, and administration was left to the discretion of the anesthesiologists. The Apfel score [9, 10] includes four variables (female sex, non-smoking status, history of PONV or motion sickness, and use of postoperative opioids) that are assigned one point each, and the total score was used to evaluate the risk of developing PONV. An Apfel score of 0 or 1 was considered low risk; 2, medium risk; and ≥ 3, high risk [1].

### Patients

Female patients who underwent obstetric and gynecologic surgery under general anesthesia, excluding emergency surgery, were investigated. Patients who underwent surgery between August 2017 and January 2018, before the introduction of the criteria for intraoperative management, were placed in the “pre-criteria group,” and those who underwent surgery from November 2018 to April 2019 were placed in the “post-criteria group.” The number of patients was determined within the observation period, before and after establishing the criteria for intraoperative management.

This study was conducted retrospectively. The following clinical data were examined: age and Apfel score as patient information; disease, laparoscopic surgery, epidural anesthesia, volatile anesthetics, and duration of surgery, anesthesia duration as PONV-related factors; and the use of dexamethasone, droperidol, and metoclopramide and their doses, patient-controlled analgesia (PCA), total intravenous anesthesia (TIVA) management, and development of PONV as intraoperative measures. PONV was defined as nausea and/or vomiting that occurred immediately after the completion of surgery until the next day. The development of PONV was confirmed by the doctors, pharmacists, and nurses, who performed postoperative rounds, and ward nurses and resident pharmacists in the ward.

### Evaluation of the usefulness of the criteria for intraoperative management of PONV

The usefulness of the criteria for intraoperative management was evaluated based on changes in the implementation rate of intraoperative management and PONV incidence. The implementation rate of intraoperative management was defined as the ratio of patients who underwent intraoperative management to that of all patients. Implementation of any of the following measures was considered: intraoperative use of antiemetics (dexamethasone, droperidol, metoclopramide), mixing of droperidol into a PCA pump, or TIVA management. The number of implemented management measures was defined as the total number of measures implemented, and the unit was the number of implemented measures. PONV incidence was defined as the ratio of the number of patients who developed PONV to the number of all patients.

### Statistics

Patient background factors, implementation rate of intraoperative management, and PONV incidence were analyzed in the pre- and post-criteria groups using the χ^2^ test, Fisher’s exact test, or Wilcoxon rank sum test. The Statistical significance was set at 5% (*p* <  0.05). JMP® 11 (SAS Institute Inc., Cary, NC, USA) was used for all statistical analyses.

### Ethical considerations

This study was approved by the institutional review board of our hospital (IRB number: 20190047). This study used only existing information, and no written or oral consent was obtained from all patients. Therefore, we disclosed information about the study and guaranteed the opportunity for all patients to refuse to be included in the study.

## Results

### Patient background

Table 1 shows the background of 369 patients, 193 and 176 of whom were in placed in the pre- and post-criteria groups, respectively. The proportion of patients with an Apfel score of 2 was significantly higher and that of patients with a score of 4 was significantly lower in the post-criteria group than in the pre-criteria group. However, there was no difference in the proportion of patients with an Apfel score ≥ 2, which was the target for intraoperative management. In addition, the proportion of patients undergoing laparoscopic surgery, a risk factor for developing PONV, was significantly higher in the post-criteria group than in the pre-criteria group. The use rate of dexamethasone and droperidol in the post-criteria group was higher than that in the pre-criteria group.

**Table 1:** Patient background

Item	Pre-criteria group (*n* = 193)	Post-criteria group (*n* = 176)	*P*
Age	46 (23–91)	46 (18–80)	NS^a^)
Apfel simplified score (point)			
1	15 (7.8)	10 (5.7)	NS^b^)
2	53 (27.5)	75 (42.6)	0.002b)
3	94 (48.7)	78 (44.3)	NS^b^)
4	31 (16.1)	13 (7.4)	0.009^b^)
Disease			
Malignant neoplasm of the ovary	17 (8.8)	20 (11.4)	NS^b^)
Borderline neoplasm of the ovary	3 (1.6)	4 (2.3)	NS^c^)
Benign neoplasm of the ovary	29 (15.0)	27 (15.3)	NS^b^)
Malignant neoplasm of the uterus	19 (9.8)	26 (14.8)	NS^b^)
Benign neoplasm of the uterus	80 (41.5)	65 (36.9)	NS^b^)
Malignant neoplasm of the soft tissue and other organs	4 (2.1)	3 (1.7)	NS^c^)
Non-inflammatory disorders of the female genital tract	36 (18.7)	28 (15.9)	NS^b^)
Others	5 (2.6)	3 (1.7)	NS^c^)
Laparoscopic surgeries	97 (50.3)	127 (72.2)	< 0.0001^b^)
Epidural anesthesia	62 (32.1)	42 (23.9)	NS^b^)
Volatile anesthetics	153 (79.3)	149 (84.7)	NS^b^)
TIVA	41 (21.2)	27 (15.3)	NS^b^)
Operative duration (minutes)	155 (49–639)	186 (36–597)	NS^a^)
Anesthesia duration (minutes)	201 (86–698)	198.5 (67–657)	NS^a^)
Number of implemented measures^d^)			
1	43 (22.3)	29 (16.5)	NS^b^)
2	75 (38.9)	97 (55.1)	0.002^b^)
3	38 (19.7)	36 (20.5)	NS^b^)
4	18 (9.3)	7 (4.0)	NS^b^)
5	2 (1.0)	0 (0)	NS^c^)
Antiemetics			
Dexamethasone	130 (67.4)	152 (86.4)	< 0.0001^b^)
Droperidol	130 (67.4)	134 (76.1)	NS^b^)
Metoclopramide	32 (16.6)	14 (8.0)	0.011^b^)
Droperidol (PCA)	56 (29.0)	32 (18.2)	0.014^b^)
Dose of antiemetics (mg)			
Dexamethasone	6.6 (0–6.6)	6.6 (0–6.6)	< 0.0001^a^)
Droperidol	0.75 (0–5.0)	0.875 (0–2.5)	NS^a^)
Metoclopramide	0 (0–20)	0 (0–10)	0.012^a^)

Numerical values represent the number of patients and percentage (%)

Continuous variables are represented as median range

a) Wilcoxon rank sum test. b) Chi-squared test. c) Fisher’s exact test

d) Number of implemented management measures including the total number of the any of the following:

intraoperative antiemetics (dexamethasone, metoclopramide, droperidol), mixture of droperidol in a PCA pump, or TIVA

Units represent the number of measures

*NS* Not significant, *TIVA* Total intravenous anesthesia, *PCA* Patient controlled analgesia

### Evaluation of the usefulness of the criteria for intraoperative management of PONV

**Fig. 2:**
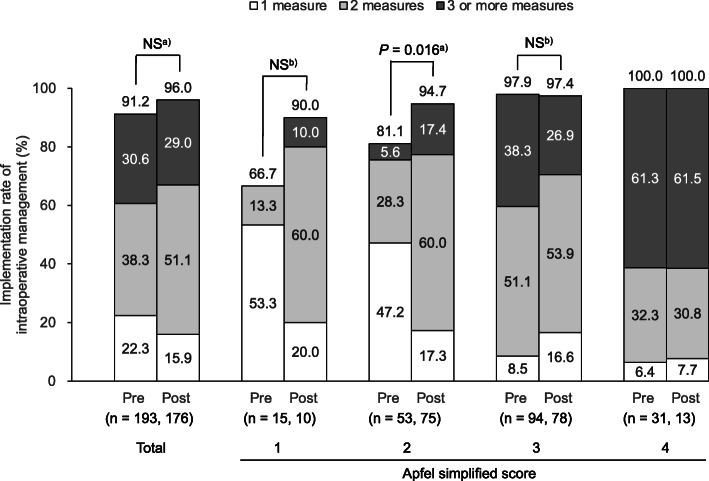
Changes in the intraoperative management implementation rate. The bar graph shows the changes in the intraoperative management implementation rate before and after the criteria in all patients and by the Apfel simplified score. **a**) Chi-squared test. **b**) Fisher’s exact test. PONV: postoperative nausea and vomiting

Figure 2 shows the changes in the implementation rate of intraoperative management of PONV. The implementation rate increased from 91.2 to 96.0% after the introduction of the criteria. The implementation rate of intraoperative management in patients with Apfel scores of 3 and 4 was similar in both pre- and post-criteria groups, but higher in patients with Apfel scores of 1 and 2. Notably, in patients with a score of 2, the rate significantly increased from 81.1 to 94.7% after the introduction of the criteria, indicating that the criteria for intraoperative management were followed. The doctor’s acceptance rate for the proposal of antiemetics was 96.4%, the ratio of patients who received antiemetics divided by the patients with an Apfel score of 2 or higher, which were the target patients for antiemetics.

**Fig. 3:**
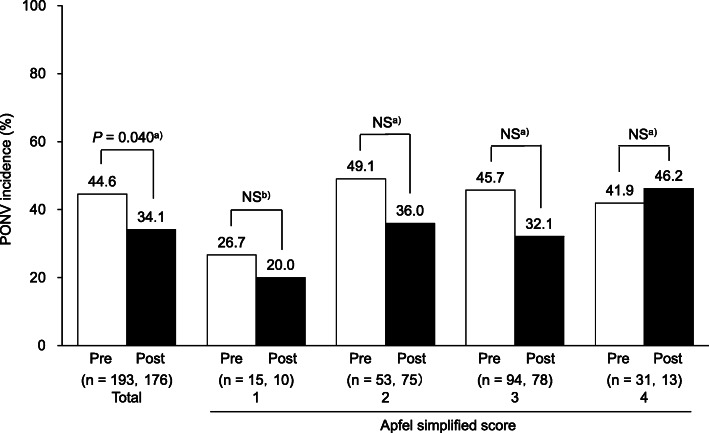
Changes in PONV incidence. The bar graph shows the changes in the incidence of postoperative nausea and vomiting (PONV) before and after the criteria in all patients and by the Apfel simplified score. Incidence of PONV in the pre-criteria (□) and post-criteria (■) groups. **a**) Chi-squared test. **b**) Fisher’s exact test.

Figure 3 shows the change in the PONV incidence. The incidence of PONV significantly decreased from 44.6 to 34.1% after the introduction of the criteria. In patients with Apfel scores of 1–3, the incidence dropped. Notably, in patients with an Apfel score of 2, the rate decreased from 49.1 to 36.0% after the introduction of the criteria.

## Discussion

In this study, pharmacists and anesthesiologists collaborated to develop criteria for intraoperative management of PONV. PONV incidence was significantly decreased by standardizing the management to improve the implementation rate of intraoperative management. Thus, it was demonstrated that the criteria for intraoperative management are useful for PONV management in female surgical patients.

The implementation rate of intraoperative management was higher in the post-criteria group than in the pre-criteria group (Fig. 2). A possible reason for this is that the establishment of the criteria for intraoperative management raised awareness among anesthesiologists for PONV management, which led to the implementation of measures in patients with an Apfel score of 1 or 2. In addition, the pharmacist-in-charge in the operating room confirmed the risk of PONV development and proposed the administration of antiemetics to the anesthesiologist, which may have contributed to the increase in the implementation rate of intraoperative management. The implementation rate of intraoperative management was similar in patients with Apfel scores of 3 and 4 in the pre- and post-criteria groups, while the rate of combination therapy (use of two or more measures) decreased in patients with an Apfel score of 3. This may be attributed to the lack of established criteria for recommending combination therapy, even in patients at high risk of developing PONV.

The incidence of PONV was significantly lower in the post-criteria group than in the pre-criteria group (Fig. 3). A reduction in PONV incidence in patients with Apfel scores of 1–3 may have contributed to this finding. In this study, there were no significant differences in patient factors that affected the development of PONV before and after the establishing the criteria for intraoperative management. The only change observed was the increase in intraoperative management implementation, which led to the reduction in PONV incidence; thus, the increase in the frequency of intraoperative management may directly affect the reduction of PONV. Unlike the previous data on Japanese patients [8], the risk of developing PONV was considered high in this study because only female patients were investigated, and the rate of the history of motion sickness and PONV was high. Under these circumstances, it is clinically significant that the incidence of PONV in women is reduced from 51.3% in a previous report [8] to 34.1% in this study by establishing the criteria for intraoperative management.

In patients with Apfel scores of 1–2, because the implementation of the two measures increased the number of implemented intraoperative management measures, the increase in the combination therapy rate was considered to have contributed to the reduction of PONV incidence. Thus far, combination therapy has been recommended only for high-risk patients with an Apfel score of ≥3 due to increased treatment costs and potential side effects [3]. However, in recent years, the use of two drugs has been recommended in patients with an Apfel score of ≥2, while the use of three or four drugs is recommended in patients with an Apfel score of at least 3 [11]; thus, combination therapy has become standardized. This indicates that management with two measures may have been effective in patients with an Apfel score of ≤2. In addition, the drugs used for combination therapy were dexamethasone and droperidol, and the implementation rate of this combination therapy was higher in the post-criteria group than in the pre-criteria group. This may have contributed to the reduction in PONV incidence after the introduction of the criteria. Although the implementation rate of intraoperative management significantly increased in patients with an Apfel score of 2, a significant decrease in PONV incidence was not observed. This may be because the proportion of patients undergoing laparoscopic surgery was 89.3% in the post-criteria group and 58.5% in the pre-criteria group, resulting in a smaller effect of intraoperative measures. In patients with an Apfel score of 3, although the implementation rate of intraoperative management was similar regardless of the criteria, the incidence of PONV was reduced. Since there was no difference in the proportion of risk factors (i.e., age, laparoscopic surgery, volatile anesthetics, and postoperative opioid analgesics), other factors that were not reviewed in this study may have been involved. Moreover, since the implementation rate of combination therapy and TIVA management decreased after the introduction of the criteria, the increase in the implementation rate of these measures may have led to a reduction in the incidence of PONV. However, PONV incidence increased in patients with an Apfel score of 4, which may have been due to a reduction in the proportion of cases with TIVA management from 48.4 to 23.1%. TIVA has been reported to exert a PONV-suppressing effect through the antiemetic effect of propofol, thus reducing the absolute risk of PONV due to volatile anesthetics by approximately 15% [12]. Therefore, TIVA management according to the risk of developing PONV may have led to a further reduction in PONV.

This study demonstrated that the standardization of management by establishing the criteria for intraoperative management reduces the incidence of PONV in women at a high risk of PONV. In addition, the study also demonstrated the possibility that establishing the criteria for intraoperative management may reduce the incidence of PONV to 30% [1, 2], which is the overseas incidence in Japan, where the number of approved drugs for PONV is limited. These were considered new findings of this study.

This study has some limitations. First, since this was a retrospective study, the number of extracted patients varied in each group before and after the measures for each Apfel score. In addition, there was a bias in the PONV development risk in the extracted patients; for these reasons, it was difficult to adjust the number of patients using the Apfel score and bias in the PONV development risk in both groups. Second, since the history of motion sickness or PONV, which was included in the variables of the Apfel score, was studied as a compound factor, it was difficult to determine the weighting of each factor’s involvement in PONV development.

## Conclusions

In this study, it was demonstrated that the introduction of criteria for the indication of intraoperative management of PONV improved the implementation rate of PONV management and significantly decreased PONV incidence in female surgical patients. This could potentially lead to reduced discomfort due to PONV development in female surgical patients and improve their satisfaction with the surgery.

However, as it was demonstrated that 3.6% of the patients who undergo intraoperative management did not receive the management, it seems necessary to establish a system in which intraoperative management is completely performed without omission. Furthermore, protocol-based drug treatment, including drug selection based on the PONV risk and recommendations for combination therapy, is considered an issue in the future.

## Data Availability

The datasets used and/or analyzed during the current study are available from the corresponding author upon reasonable request.

## Abbreviations

PONV: Postoperative nausea and vomiting
TIVA: Total intravenous anesthesia
PCA: Patient-controlled analgesia
